# Discovery, synthesis and antibacterial evaluation of phenolic compounds from *Cylicodiscus gabunensis*

**DOI:** 10.1186/s12906-019-2589-2

**Published:** 2019-07-24

**Authors:** Omar Aldulaimi, Falko Drijfhout, Fidelia I. Uche, Paul Horrocks, Wen-Wu Li

**Affiliations:** 1 School of Pharmacy and Bioengineering, Keele University, Stoke-on-Trent, ST4 7QB UK; 2 College of Pharmacy, Al-Mustansiriyah University, Baghdad, Iraq; 3 Chemical Sciences Research Centre, Keele University, Staffordshire, ST5 5BG UK; 4 Present address: School of Computing, Engineering and Physical Sciences, University of the West of Scotland, High Street, Paisley, PA1 2BE UK

**Keywords:** *Cylicodiscus gabunensis*, *Leguminosae*, Antibacterial activity, Alkyl gallates, Scanning electron microscopy

## Abstract

**Background:**

*Cylicodiscus gabunensis* Harms (Family *Leguminosae*) (CG) is an African medicinal plant used as a treatment of various ailments including malaria, liver diseases, and gastrointestinal disturbances. Its extracts showed potent in vitro antibacterial activity. However, the antibacterial components are unknown.

**Methods:**

In this study, the stem bark of the CG plant was extracted and its antibacterial property against a panel of Gram-negative and Gram-positive bacterial strains assessed using the disk diffusion assay method. Bioassay-guided fractionation of the bioactive extracts was employed to identify bioactive constituents using both gas and liquid chromatography mass spectrometry. Chemical synthesis was used to make the analogues of gallic acid. Microplate dilution assays and scanning electron microscopy (SEM) were used to evaluate the antibacterial properties and mechanism of action of the active fractions and pure compounds.

**Results:**

The most bioactive sub-fractions derived from CG comprised of ethyl gallate, gallic acid and polyphenols. Five alkyl/alkenyl gallates were synthesized. A preliminary structure-activity relationship of gallic acid derivatives was obtained using the synthetic analogues and a series of commercially available phenolic compounds. Increasing the length of alkyl chains generally increases the potency of the alkyl gallates. Introducing a double bond with restricted conformations of the C-5 side chain has little effect on the antibacterial property. SEM analysis of the effect of alkyl gallates on *Staphylococcus aureus* indicates that they appear to interrupt *S. aureus* bacterial cell wall integrity.

**Conclusions:**

The results of this research rationalise the ethnobotanical use of *C. gabunensis* and suggest that gallate derivatives may serve as promising antibacterial agents for the treatment of infectious diseases.

**Electronic supplementary material:**

The online version of this article (10.1186/s12906-019-2589-2) contains supplementary material, which is available to authorized users.

## Background

Plants have been used by mankind to satisfy their medicinal needs from time immemorial. The plant kingdom produces a diverse range of secondary metabolites with a plethora of chemical scaffolds that have been optimized to possess biological activity [1]. About 120 plant-derived active compounds are medically in use, comprising about 25% of the globally prescribed drugs. Furthermore, 11% of the total 252 drugs in the WHO’s essential medicine list by 2011 are of plant origin [2]. In the process of drug discovery, plant screening based on ethno-pharmacological claims has been reported with a high success rate [1]. Secondary metabolites from medicinal plants have been shown to be a good source of antimicrobials when used alone or in combination with antibiotics to fight life threatening microbes [3–5].

*Cylicodiscus gabunensis* Harms (CG) (*Leguminosae*) is an indigenous medicinal plant widely distributed in West and Central Africa. Its bark extract has been used as an analgesic, antipyretic, anti-inflammatory herbal drug, and for the treatment of jaundice and malaria by the Ibibio of Niger Delta region of Nigeria [6, 7]. Aqueous CG extracts have recently showed antioxidant and anti-diabetic properties [8, 9]. So far, triterpenes such as cylicodiscic acid [10], triterpenoid saponins [11–13], cyclodione [14], and coumestan glycosides [15] have been isolated and identified from this plant. We have recently characterized the most antimalarial components of CG extracts as a mixture of phenolic acid and polysaccharide conjugates [16] providing evidence for its use by the Ibibio people of Nigeria as antimalarial remedy [17]. The ethyl acetate extract from CG also shows broad antibacterial activities against several clinical isolates such as *Escherichia coli*, *Staphylococcus aureus*, and *Pseudomonas aeruginosa* [7]; however, the source of these antibacterial activities have not yet been determined. In continuation of our search for antibacterial agents from natural resources [18–20], here we report the bioassay-guided fractionation, isolation, identification, synthesis, and evaluation of antibacterial activity of compounds from CG bark extract. A systematic study of the structure activity relationship of synthetic and commercially available alkyl gallates was also carried out.

## Methods

### Plant materials and chemicals

*C. gabunensis* stem bark was collected in September 2012 from the woods of Imo state, Nigeria and authenticated by H. Donyeachusim, a taxonomist in the University of Port Harcourt, Nigeria. A voucher specimen is deposited in the University of Port Harcourt, Nigeria (UPH1028). All the solvents used in this study supplied by Fischer Scientific, UK are of high analytical grade and HPLC grade when used for chromatography. 3,4,5-trihydroxybenzoic acid (gallic acid, **1**); 3,4-dihydroxybenzoic acid (protocatechuic acid, **2**); 4-hydroxy-3-methoxybenzoic acid (vanillic acid, **3**); 4-hydroxy-3,5-dimethoxybenzoic acid (syringic acid, **4**); 3,4,5-trimethoxybenzoic acid (**5**); ethyl 3,4,5-trihydroxybenzoate (ethyl gallate, **6**); propyl 3,4,5-trihydroxybenzoate (propyl gallate, **7**); and dodecyl 3,4,5-trihydroxybenzoate (lauryl gallate, **13**) were purchased from Sigma Aldrich UK.

### Preparation of extracts and fractions

The extraction of CG bark (120 g) was done according to a general procedure [21] for the extraction of secondary metabolites such as saponins which were previously reported in this plant [11–13]. The plant bark powder was first defatted using hexane to give the nonpolar hexane extract (CGH, 0.9 g, 0.75% *w/w*). The residue was extracted again using 70% ethanol twice to give the polar ethanol extract (CGE, 11.3 g, 9.4% *w/w*) (Additional file 1: Figure S1) [16]. CGE was further partitioned in water with ethyl acetate and n-butanol sequentially [21] to give the ethyl acetate fraction (CGEEA, 0.9 g, 8% *w/w*), butanol fraction (CGEBU, 8.3 g, 77% *w/w*) and aqueous fraction (CGEAQ, 1.6 g, 15% *w/w*).

### Purification and fractions of CGEEA fraction

The CGEEA fraction (0.85 g) was fractionated on a silica gel column (35–75 μm; 2.5 × 50 cm) eluting with hexane: ethyl acetate (4:1), ethyl acetate and finally methanol. Each fraction was 100 mL and 18 fractions were collected. The detection of eluates was performed using thin layer chromatography (SiO_2_, the solvent system is a mixture of hexane and ethyl acetate (3:2)) and visualized under UVGL-25 compact UV lamp at 254/365 nm. All the fractions were checked by TLC and the fractions with similar compound patterns were combined and dried at 45 °C under vacuum, yielding 8 combined fractions, CGEEA-F1-8: F1, 29 mg; F2, 9.1 mg; F3, 8.8 mg; F4, 8.9 mg; F5, 450 mg; F6, 130 mg; F7, 72.3 mg; and F8, 97.2 mg.

### Purification of CGEEA-F5 using high performance liquid chromatography

An Agilent 1200 series high performance liquid chromatography (HPLC) system consisting of G2258A DLA dual loop auto-sampler and G1361A preparative pump were used for the further purification of CGEEA-F5 (0.42 g) using a X bridge C18 Prep column (5 μm, 19 mm × 100 mm) and monitored at 254 nm using a Waters G1315D DAD Diode Array Detector. Solvents (A: 0.1% *v/v* glacial acetic acid in water, B: 0.1% *v/v* glacial acetic acid in methanol) were used at a flow rate of 16 mL/min and a max pressure of 400 bar. A gradient of A and B solvent system (5% B for 5 min; 5–60% B over 25 min; and 100% B for 6 mins) was used. Twelve sub-fractions (CGEEA-F5-(1 to 12)) were collected and lyophilized to give: F1, 12 mg; F2, 2 mg; F3, 2 mg; F4, 2 mg; F5, 2 mg; F6, 4 mg; F7, 16 mg; F8, 21 mg; F9, 83 mg; F10, 223 mg; F11, 13 mg; and F12, 8 mg.

### Gas chromatography mass spectrometry

The chemical constituents of CGEEA-F5, CGEEA-F5–8 and synthetic gallate derivatives (**8**–**12**) after deriving into their corresponding trimethylsilyl (TMSi) derivatives were characterized using gas chromatography mass spectrometry on HP5-MS column as previously described [16, 22].

### Time-of-flight liquid chromatography mass spectrometry (TOF LC-MS)

The Infinity 1260 series system comprises a G4225A Hip Degasser, G1312B Binary Pump, G1329B ALS Auto sampler, G1316A TCC Thermostated column compartment, HyPURITY C8 column (50 × 2.1 mm, particle size 5 μm) from Thermo Fisher Scientific, UK and 6530 Accurate Mass Q-TOF as the detector. Qualitative analysis Mass Hunter work station software, ion Source Dual ESI, ion Polarity positive, a detector with mass range 100–1700 m/z were used throughout. A flow rate of solvent (A: 0.1% formic acid in water, B: acetonitrile) was kept at 0.5 ml/min with a gradient of keeping 5% B over 10 min and increasing from 5 to 95% from 10 to 18 min. Molecular formulae were generated using high resolution mass by consideration of likelihood of the unsaturation degrees using Mass Hunter work station software. The putative identification of polyphenols (Table 2) was performed by searching the obtained molecular formula against the ChemSpider database of the Royal Society of Chemistry (http://www.chemspider.com) and Reaxy database (www.reaxy.com).

### Analysis of CGEEA-F5 and CGEEA-F5–8 by analytical HPLC

Analysis of CGEEAF-5 and CGEEAF-5-8 was carried out using a linear gradient from 10 to 70% B over 25 mins and keeping 100% B for 6 mins, where A was water and B methanol on an analytical HPLC column (Phenomenex, UK; Jupiter C18 reverse phase column, 300 A, 5 μm particle size, 4.6 mm × 250 mm) at a flow rate of 1 mL/min. Gallic acid and ethyl gallate were used as internal standards.

### Synthesis of alkyl and alkenyl 3, 4, 5-trihydroxybenzoates (gallic acid derivatives)

A general procedure of synthesis of gallate derivatives (**8**–**12**) was based on a previous report [16, 30] and described as follows with modification. To a solution of gallic acid (3.0 mmol; 510 mg) and anhydrous alcohols (i.e. isopropanol, 2-methylpropanol, 2-methylbutanol, 3-methylbutanol, or 3-methylbutenol, each 4.0 mmol) in tetrahydrofuran (THF) (6 ml) in an ice bath at 0 °C, a solution of N,N-dicyclohexylcarbodiimide (DCC) (3.5 mmol, 720 mg) in THF (6 ml) was added. The mixture was allowed to stir for 24 h, and was evaporated under vacuum. The product, a dark yellow residue, was extracted with cold ethyl acetate three times. The upper layers were combined and evaporated. The synthesized esters were purified using silica gel column chromatography (35–75 μm; 4 × 30 cm) by eluting with hexane: ethyl acetate (7: 3, isocratic) at a flow rate of 3 ml/min. The eluates were subjected to thin layer chromatography (silica gel, ethyl acetate: hexane 7: 3) and visualised under a UV lamp (254/365 nm) or exposed to iodine vapour.

Propan-2-yl 3,4,5-trihydroxybenzoate (**8,** 534 mg, 83%): positive-ion TOF LC-MS, *m/z*: 235.0576 (calcd. mass for C_10_H_12_NaO_5_, [M + Na]^+^, 235.0582). GC-EI-MS of TMSi derivative of **8** (Rt, 19.141 min), *m*/*z* (%): 428.2 (40), 369.2 (5), 311.1 (6), 281.1 (55), 239.0 (35), 209.0 (5), 179.0 (9), 133.0 (7), 73.1 (100). ^1^H NMR (400 MHz, CD_3_OD) *δ*: 7.06 (2H, s, H-2, 6), 5.09–5.17 (1H, m, H-8), 1.34 (6H, d, *J* = 6.4 Hz, H-9, 10). ^13^C NMR (100 MHz, CD_3_OD) *δ*: 166.7 (C-7), 145.0 (C-3, 5), 138.2 (C-4), 120.8 (C-1), 108.6 (C-2, 6), 67.8 (C-8), 20.8 (C-9, 10).

2-Methylpropyl 3,4,5-trihydroxybenzoate (**9,** 583 mg, 85%): positive-ion TOF LC-MS, *m/z*: 227.0917 (calcd. mass for C_11_H_15_O_5_, [M + H]^+^, 227.0919). GC-EI-MS of TMSi derivative of **9** (Rt, 20.592 min), *m*/*z* (%): 442.3 (45), 369.2 (8), 281.1 (55), 239.1 (33), 179.0 (5), 133.0 (5), 73.1 (100). ^1^H NMR (400 MHz, CD_3_OD) *δ*: 7.08 (2H, s, H-2, 6), 4.02 (2H, d, *J* = 6.6 Hz, H-8), 2.04 (1H, dt, *J* = 13.3, 6.6 Hz, H-9), 1.03 (6H, d, *J* = 6.6 Hz, H-10, 11). ^13^C NMR (100 MHz, CD_3_OD) *δ*: 167.2 (C-7), 145.1 (C-3, 5), 138.3 (C-4), 120.3 (C-1), 108.6 (C-2, 6), 70.3 (C-8), 27.8 (C-9), 18.1 (C-10, 11).

2-Methylbutyl 3,4,5-trihydroxybenzoate (**10**, 597 mg, 82%): Positive-ion TOF LC-MS, *m/z*: 241.1073 (calcd. mass for C_12_H_17_O_5_ [M + H]^+^, 241.1076). GC-EI-MS of TMSi derivative of **10** (Rt, 21.440 min), *m*/*z* (%): 456.3 (55), 369.2 (8), 311.1 (4), 281.1 (55), 239.0 (25), 179.0 (10), 133.0 (5), 73.1 (100). ^1^H NMR (400 MHz, CD_3_OD) δ: 7.07 (2H, s, H-2, 6), 4.02–4.16 (2H, m, H- 8), 1.79–1.87 (1H, m, H-9), 1.45–1.55 (1H, m, H-10), 1.26–1.34 (1H, m, H-10), 1.02 (3H, d, *J* = 6.8 Hz, H-12), 0.95–1.01 (3H, t, H- 11). ^13^C NMR (101 MHz, CD_3_OD) δ: 167.2 (C-7), 145.1 (C-3, 5), 138.3 (C-4), 120.3 (C-1), 108.6 (C-2, 6), 68.8 (C-8), 34.3 (C-9), 25.8 (C-10), 15.5 (C-12), 10.3 (C-11).

3-Methylbutyl 3,4,5-trihydroxybenzoate (**11**, 604 mg, 83%): positive-ion TOF LC-MS, *m/z*: 241.1071 (calcd. mass for C_12_H_17_O_5_, [M + H]^+^, 241.1071). GC-EI-MS of TMSi derivative of **11** (Rt, 21.396 min), *m*/*z* (%): 456.3 (55), 369.2 (8), 311.1 (4), 281.1 (55), 239.0 (25), 179.0 (10), 133.0 (5), 73.1 (100). ^1^H NMR (400 MHz, CD_3_OD) δ: 7.06 (2H, s, H- 2, 6), 4.27 (2H, t, J = 6.6 Hz, H- 8), 1.64 (2H, q, J = 6.7 Hz, H- 9), 1.25 (1H, m, H- 10), 0.99 (6H, d, J = 6.6 Hz, H- 11, 12). ^13^C NMR (100 MHz, CD_3_OD) *δ*: 167.2 (C-7), 145.1 (C-3, 5), 138.3 (C-4), 120.3 (C-1), 108.6 (C-2, 6), 62.8 (C-8), 37.3 (C-9), 25.0 (C-10), 21.5 (C-11, 12).

3-Methylbut-2-en-1-yl 3,4,5-trihydroxybenzoate (**12**, 606 mg, 84%): Positive-ion TOF LC-MS, *m/z*: 239.0922 (calcd. Mass for C_12_H_15_O_5_, [M + H]^+^, 239.0919). GC-EI-MS of TMSi derivative of **12** (Rt, 21.798 min), *m*/*z* (%): 454.2 (35), 386.2 (12), 355.1 (6), 311.1 (4), 281.1 (55), 239.1 (25), 179.0 (10), 133.0 (5), 73.1 (100). ^1^H NMR (400 MHz, CD_3_OD) *δ*: 7.05 (2H, s, H-2, 6), 5.45 (1H, ddt, *J* = 8.6, 5.8, 1.4 Hz, H-9), 4.74 (2H, d, *J* = 7.2 Hz, H-8), 1.79 (6H, d, *J* = 2.4 Hz, H-11, 12). ^13^C NMR (100 MHz, CD_3_OD) *δ*: 167.1 (C-7), 145.1 (C-3, 5), 138.6 (C-10), 138.3 (C-4), 120.4 (C-1), 118.7 (C-9), 108.6 (C-2, 6), 61.0 (C-8), 16.7 (C-11), 24.5 (C-12).

#### Bacterial strains and growth conditions

Bacterial isolates were supplied by the National Collection of Industrial and Marine Bacteria NCIMB Ltd., Aberdeen, Scotland. The bacterial isolates used were *Bacillus cereus* NCIMB 9945, *Bacillus subtilis* NCIMB 3610, *Staphylococcus aureus* NCIMB 11832, *Staphylococcus epidermidis* NCIMB8853, *Enterobacter cloacae* NCIMB 10101, *Escherichia coli* NCIMB8277, *Alcaligenes faecalis* NCIMB 8156, *Pseudomonas aeruginosa* NCIMB 10848 and *Enterococcus faecalis* NCIMB 2707. For the disk diffusion assay, the inoculum density used was 1.5 × 10^8^ CFU/ mL with 100 μL of inoculum plated on bacterial nutrient broth (Oxoid, UK).

### Disk diffusion assay

Disk diffusion assay (DDA) was run according to the EUCAST standards [31]. CGH and CGE extracts and CGEEA, CGEBU and CGEAQ fractions were dissolved in dimethyl sulfoxide (DMSO). The CGH and CGE extracts were prepared in four different concentrations as 100, 50, 25, and 12.5 mg/mL. The CGEEA, CGEBU and CGEAQ fractions were prepared as 12.5 mg/mL. 20 μL of each preparation were distributed evenly on Whatman sterile filter paper disks (6 mm, Fischer Scientific, UK) to yield 2.0 mg/disk, 1.0 mg/disk, 0.5 mg/disk, and 0.25 mg/disk. Two controls were used on each plate; for the first was the carrier solvent DMSO (final concentration, 3% *v/v*) as negative control and the second ampicillin as positive control with an amount of 10 or 2 μg per disk. The disks were applied firmly onto the surface of the inoculated agar plate, inverted and incubated at 37 °C overnight in a LMS series incubator. The recorded inhibition zone (IZ) is an indication of the antibacterial activity which increases in size as the potency of the compound increases. All experiments were carried out using three biological replicates with the mean IZ and standard deviation (SD) reported.

### Determination of minimum inhibitory concentration

The minimum inhibitory concentrations (MICs) of the plant extracts and compounds explored in this study were determined using the microplate Alamar Blue assay as previously described [32, 33]. The bacterial isolates were first adjusted to McFarland standard 0.5 (~ 1 × 10^8^ CFU/ml), which were then diluted by adding 0.2 ml bacterial suspension to 19.8 ml sterile broth medium (1:100 fold dilution) to give the final bacterial concentration (1× 10^6^ CFU/ml).

Briefly, 100 μL of each bacterium culture with a bacterial density of 1 × 10^6^ CFU/mL was distributed into each test well and growth control well in black 96-well microplates (Fischer-Scientific, UK). The tested materials were dissolved in 6% DMSO (*v/v*) to provide a maximum concentration of 3% (*v/v*) DMSO and a minimum concentration at 0.023% (*v*/*v*). Ampicillin was included as a positive control at a maximum concentration of 64 μg/mL and a minimum concentration of 0.5 μg/mL. The final concentrations of  the  extracts/pure compounds were tested from 8 to 1024 μg/mL. After 24 h of incubation at 37 °C, 20 μL of Alamar Blue solution (Fischer Scientific, UK) was added to each well and the plates incubated for 2–4 h at 37 °C to enable the colour reaction to develop. A GloMax-plus®-Multi Detection System with a fluorescent filter (Ex 525 nm and Em 580–640 nm) was utilised to determine the fluorescence in each well. Data presented represents two technical replicates as two biological replicates (*n* = 4). The following equation was used to calculate the percentage of inhibition for each drug used: [1 – (FU_test well_ – FU_SC well_) / (FU_CG well_ – FU _SC well_)] × 100, where FU_test well_ represents mean of fluorescence in the absence of ampicillin/extract; FU_SC well_, mean of fluorescence in the sterility control; FU _GC well_, mean of fluorescence in growth in wells with CG extract. The MICs were determined as the lowest concentration that produced a decrease in bacterial population by 90% [32].

### Scanning electron microscopy

Gallic acid (**1**), ethyl gallate (**6**), propan-2-yl 3,4,5-trihydroxybenzoate (**8**), and 2-methylpropyl 3,4,5-trihydroxybenzoate (**9**) were added separately into 3 mL of *S. aureus* culture at a concentration that represented 2 × MIC (Table 1). The bacterial culture was used at 1.0 × 10^8^ CFU/mL and a carrier solvent control (0.2% DMSO-treated) sample was studied in parallel. After 1 h of contact with the compounds, the specimens were prepared for studying by scanning electron microscopy (SEM) as described [34]. The specimens were coated with a thin gold film using an Emscope FD 500 sputter coater before imaging using the Scanning Electron Microscope (Model S-4500, Hitachi, Japan).

**Table 1:** The MIC (μg/ml) of CG sub-fractions, synthetic and commercially sourced gallate derivatives against a panel of six bacteria

Fractions/pure compound	*S. aureus*	*S. epidermidis*	*E. coli*	*B. subtilis*	*B. cereus*	*A. faecalis*
CGEEA-F5	256	128	1024	> 1024	1024	256
CGEEA-F5–8	128	64	512	NA	NA	128
3,4,5-Trihydroxybenzoic acid (gallic acid, **1**)	2000	500	1000	1000	1000	1000
3,4-Dihydroxybenzoic acid (**2**)	1000	500	1000	1000	1000	500
4-Hydroxy-3-methoxybenzoic acid (**3**)	1000	1000	1000	1000	1000	500
4-Hydroxy-3,5-dimethoxybenzoic acid (**4**)	1000	1000	500	1000	2000	500
3,4,5- Trimethoxybenzoic acid (**5**)	1000	1000	1000	1000	1000	500
Ethyl 3,4,5-trihydroxybenzoate (ethyl gallate, **6**)	1000	250	62.5	1000	1000	250
Propyl 3,4,5-trihydroxybenzoate (**7**)	500	250	125	1000	1000	250
Propan-2-yl 3,4,5-trihydroxybenzoate (**8**)	1000	250	62.5	>1000	>1000	250
2-Methylpropyl 3,4,5-trihydroxybenzoate (**9**)	500	250	500	1000	1000	500
2-Methylbutyl 3,4,5-trihydroxybenzoate (**10**)	250	250	250	250	500	250
3-Methylbutyl 3, 4, 5-trihydroxybenzoate (**11**)	250	250	250	250	500	500
3-Methylbut-2-en-1-yl 3,4, 5-trihydroxybenzoate (**12**)	250	250	500	500	1000	500
Dodecyl 3,4,5-trihydroxybenzoate (lauryl gallate, **13**)	8	8	>1000	31.25	31.25	1000
Ampicillin	≤0.5	≤0.5	≤0.5	≤0.5	>64	16

*NA* not assayed

## Results

### Disk diffusion assay of extracts and fractions

The extraction and bioassay-guided fractionation of CG was carried out using a scheme reported in Additional file 1: Figure S1 with the antibacterial activity of the CG extracts and their fractions initially assayed using the DDA method (Additional file 1: Figure S2). The polar extract of CG (CGE) showed more potent activity than CGH against *S. aureus*, *S. epidermidis*, *B. cereus*, *B. subtilis* and *S. facecalis*. CGH showed no effect against *S. epidermidis*, *B. cereus*, *B. subtilis* and *S. facecalis* and only mild effect against *S. aureus* (Additional file 1: Tables S1 and S2). The largest IZ for CGE were recorded as 19.5 ± 1.0 and 16.5 ± 0.5 mm against *S. epidermidis* and *S. aureus*, respectively. The CGEEA, CGEBU, and CGEAQ all showed moderate to good antibacterial activities against the gram-positive strains but not the gram-negative strains except *Alcaligenes faecalis* (Additional file 1: Table S1).

### Antibacterial activity of CGEEA fractions and sub-fractions

CGEEA was separated further into eight sub-fractions using silica gel column chromatography. These sub-fractions were assayed for their inhibition of six bacterial species against which CGEEA had shown the most potent activity in the DDA assays (data not shown). Fraction 5, termed CGEEA-F5, was the most active fraction (Table 1). Preparative HPLC (Additional file 1: Figure S3) was used to separate fraction CGEEA-F5 into 12 further fractions, which were also tested using the microdilution assay. Sub-fraction 8, termed CGEEA-F5–8, was the most active sub-fraction, showing improved potency against *S. aureus* and *S. epidermis* with MICs of 128 and 64 μg/ml, respectively (Table 1 and Additional file 1: Table S3).

### Characterisation of bioactive compounds

**Fig. 1:**
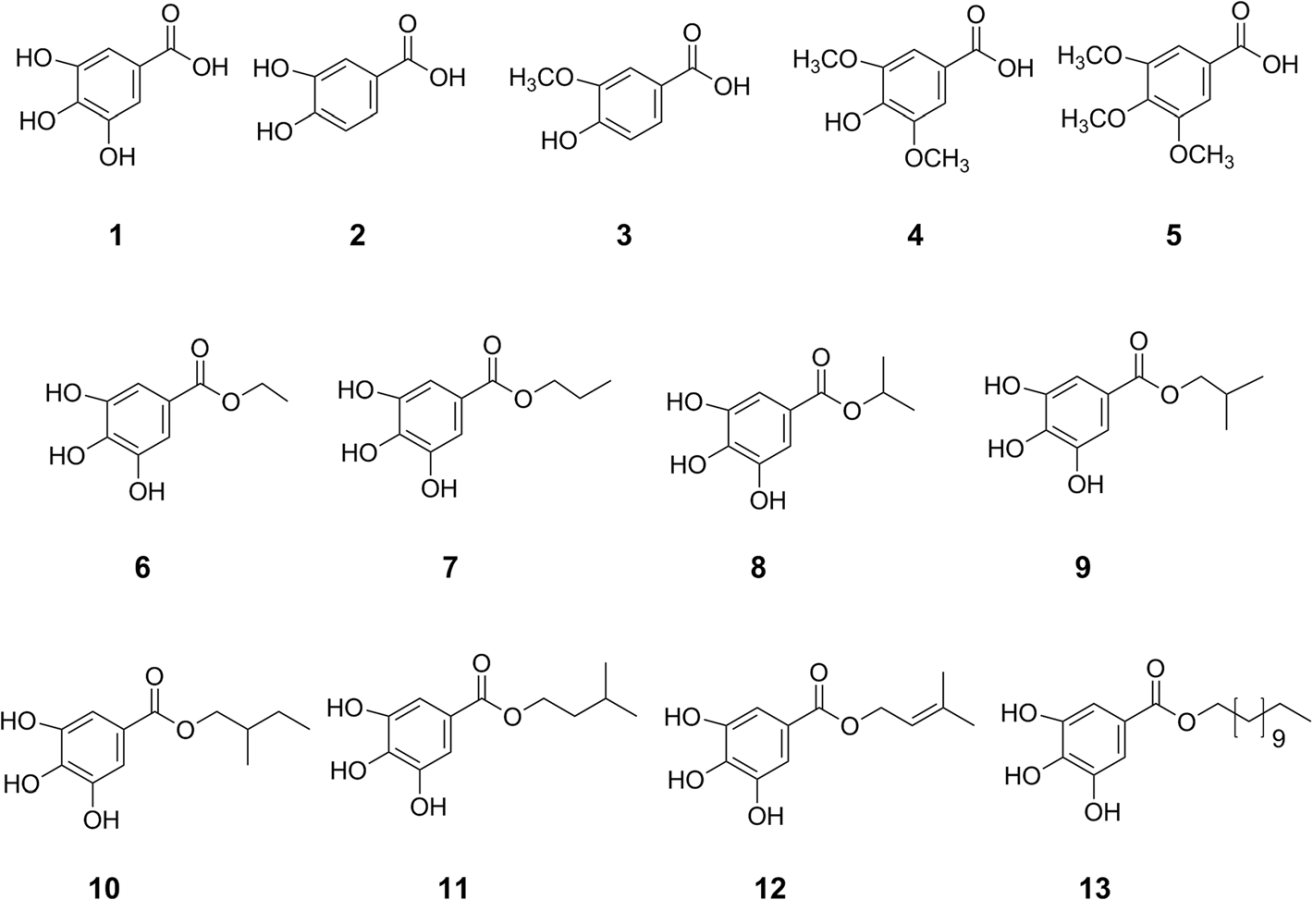
Chemical structure of gallic acid derivatives isolated from *C. gabunensis*, synthetic and commercially available gallate derivatives used in this study

CGEEA-F5 (Additional file 1: Figure S4) and CGEEA-F5–8 (Additional file 1: Figure S5 and S6) were characterized by gas chromatography mass spectrometry (GC-MS). A number of fatty acids, sugars, and phenolic derivatives such as gallic acid (**1**), syringic acid (**4**), and ethyl gallate (**6**) (Fig. 1, Additional file 1: Table S4) were identified in CGEEA-F5. GC-MS analysis of CGEEA-F5–8 revealed the presence of abundant ethyl gallate (**6**), with only traces of gallic acid and syringic acid (Additional file 1: Figure S5 and S6). The presence of these components was confirmed using analytical HPLC with commercially sourced materials as standards (Additional file 1: Figure S7). Liquid chromatography electrospray ionization high resolution mass spectrometric (ESI-MS) analysis of CGEEA-F5–8 further confirmed the presence of ethyl gallate and syringic acid (Additional file 1: Figure S8). Several other compounds with high molecular weights were tentatively identified as 3-(4-hydroxybenzoyl)epicatechin, phenolic glycosides, and condensed tannins (proanthocyanidins) (Table 2) by high resolution mass spectrometry and ^1^H NMR spectroscopy (Additional file 1: Figure S9). These compounds were unlikely detected using GC-MS due to a mass limit (1050 Da) of detection and low volatility of their TMSi derivatives.

**Table 2:** Tentative identification of compounds detected from CGEEA-F5–8 by LC-MS/MS using on-line ChemSpider database (http://www.chemspider.com) and Reaxys database (https://www.reaxys.com)

RT (min)	Molecular Formula	Molecular mass	Found [M + H]^+^ *m/z*	Name of compound	Chemical structure	Reference
2.36	C_7_H_6_O_5_	170.0214	171.0287	Gallic acid (**1**)		[16]
2.36	C_9_H_10_O_5_	198.0528	199.0603	Ethyl gallate (**6**)		[16]
3.07	C_20_H_22_O_9_	406.1265	407.1338	Trans-3,5,3′,4′-tetrahydroxystilbene-3-O-β-D-glucopyranoside		[23]
3.44	C_22_H_26_O_13_	498.1373	499.1446	3,4,5-Trimethoxyphenyl 6-O-(3,4,5-trihydroxybenzoyl)-β-D-glucopyranoside		[24]
3.45	C_22_H_18_O_8_	410.1001	411.1074	3-(4-Hydroxybenzoyl)epicatechin		[25]
3.68	C_37_H_30_O_14_	698.1639	699.1713	Epicatechin-(4β → 8)-catechin-3-O-(4-hydroxy)benzoate		[26]
3.72	C_37_H_30_O_15_	714.1585	715.1658	Epiafzelechin-(4β → 8)- epicatechin-3-O-gallate		[27]
3.86	C_45_H_38_O_16_	834.2151	835.2223	Procyanidin C-1		[28]
3.67	C_45_H_38_O_17_	850.2104	851.2177	Epicatechin--(4β → 8)-epicatechin--(4β → 8)-catechin trimer		[29]

### Antibacterial activity of isolated and synthetic phenolic compounds

To gain structure activity relationship of gallate and alkyl gallate derivatives, five gallate analogues (**8**–**12**) were synthesized. The MICs of gallic acid (**1**), ethyl gallate (**6**), the synthetic (**8**-**12**) and commercially available (**2**–**5**, **13**) gallate analogues (Table 1, Fig. 1) were determined. Ethyl gallate (**6**) is more potent than gallic acid in all of the six bacteria species tested. In particular, **6** showed potency against *S. epidermidis* and *E. coli.* However, it showed less potency against *S. aureus* than *S. epidermidis*, whilst CGEEA-F5–8, containing both ethyl gallate and gallic acid, showed strong inhibition. This may be that additional polyphenols are more active, or potentiate the activity of ethyl gallate and/or gallic acid.

The effect of alkyl gallates was more pronounced against both *S. aureus *and *S. epidermidis  *species tested. *S. epidermidis* was more sensitive to the compounds used, with the order of activity as follows: vanillic acid (**3**), syringic acid (**4**), and 3,4,5-trimethoxybenzoic acid (**5**) < gallic acid (**1**), protocatechuic acid (**2**) < ethyl gallate (**6**), propyl gallate (**7**), isopropyl gallate (**8**), **9**–**12** < lauryl gallate (**13**). Strikingly, lauryl gallate (**13**) showed the most potent effect against both *S. aureus* and *S. epidermidis* (MIC ≤8 μg/ml). These results suggest that the alkyl chain length of these gallate derivatives is an important factor contributing to the antibacterial activities against *S. aureus* and *S. epidermidis*. In general, the phenolic hydroxyls show the same, or reduced potency against *S. aureus* compared to *S. epidermidis*.

### Scanning electron microscopy

Scanning electron microscopy (SEM) was used to study the ultrastructure of *S. aureus* cell wall integrity following exposure to gallic acid (**1**), ethyl gallate (**6**), propan-2-yl 3,4,5-trihydroxybenzoate (**8**) and 2-methylpropyl 3,4,5-trihydroxybenzoate (**9).**

**Fig. 2:**
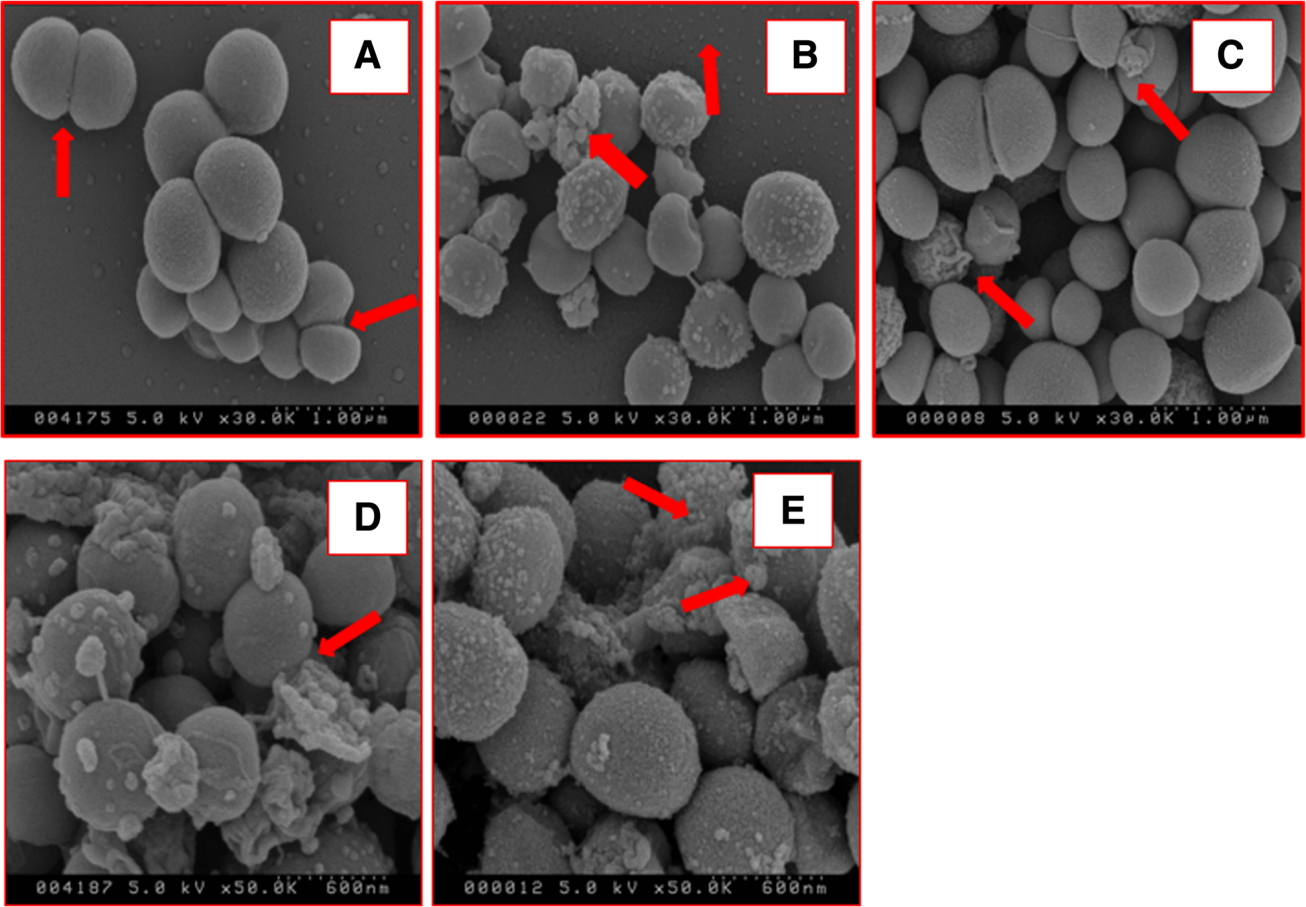
Scanning electron micrographs (SEM) of *S. aureus* exposed to gallate derivatives. **a**) Control SEM of *S. aureus* cells after exposure to 0.2% DMSO for 1 h with arrows pointing to cells undergoing binary fission. SEM of *S. aureus* cells after exposure to 2 × MIC level of **b**) gallic acid (**1**), **c**) ethyl gallate (**6**), **d**) propan-2-yl 3,4,5-trihydroxybenzoate (**8**), and **e**) 2-methylpropyl 3,4,5-trihydroxybenzoate (**9**). For panels B to E arrows point to apparent extrusion of cytoplasmic content from collapsed *S. aureus*

For all of these phenolics, when compared to an untreated control (Fig. 2a), all treatments affected *S. aureus* cell wall integrity (Fig. 2b-e) with evidence of loss of cell shape and leakage of cytoplasmic content onto the surface of the bacteria.

Propan-2-yl 3,4,5-trihydroxybenzoate (**8**) and 2-methylpropyl 3,4,5-trihydroxybenzoate (**9**) appear to cause a more prominent effect (Fig. 2d-e) with a greater proportion of damaged *S. aureus* evident and more evidence of extruded cytoplasmic material, when compared to gallic acid and ethyl gallate. As such, these SEM data support that alkyl gallates are apparently more potent in agreement with the MIC data (Table 1).

Esterification of gallic acid with alkyl group with five and twelve carbon chains (**10**, **11**, and **13**) also increased the antibacterial activity against *B. cereus* and *B. subtilis* compared to gallic acid and other alkyl gallates with shorter carbon chains (Table 1). 3-Methylbut-2-en-1-yl 3,4,5-trihydroxybenzoate (**12**) with a double bond with restriction of the conformational change of the side chain led to a 2-fold decrease of the antibacterial effect against *E. coli*, *B. cereus* and *B. subtilis* compared to 3-methylbutyl 3,4,5-trihydroxybenzoate (**11**), but no effect on other bacteria tested. Lauryl gallate (13) also showed more potent activity against *B. cereus* and *B. subtilis*. However, in a reverse of this trend, ethyl gallate (**6**) and isopropyl gallate (**8**) were more potent against *E. coli* than lauryl gallate (**13**).

## Discussion

Previously, the antibacterial activity of CG extracts was evaluated. However, the bioactive constituents responsible for the observed biological activity are unknown [7]. CGEEA was the most potent among the CGE sub-fractions, in agreement with the previous report [7] with the exception of *P. aeruginosa* shown as inactive here. Our results demonstrated the presence of various phenolic compounds including gallate derivatives and complex polyphenols in the CG extracts using bioassay-guided fractionation, GC-MS, LC-MS and NMR characterization. Gallic acid, ethyl gallate and alkyl gallates were also found to have anti-plasmodial activity in our previous study [16]. Surprisingly, no previously reported triterpenoids [10] and saponins [11–13] were found in these antibacterial fractions.

There have been a number of studies describing the antibacterial and antifungal effect of alkyl gallates [30, 35, 36]. The esterification of gallic acid also increases antifungal activity, with MIC of 12.5 μg/ml for lauryl (C-12) gallate (**13**) where both the alkyl group and catechol or pyrogallol groups are essential for this antifungal activity [30]. Lauryl gallate (**13**) exhibits bactericidal activity against methicillin resistant *S. aureus* (MRSA) strains, with the minimum bactericidal concentration of 25 μg/mL due to its ability to inhibit respiratory electron transport systems [37]. Our results showed that lauryl gallate has weak effect against *E. coli* and this could be attributed to the nature of the gram-negative *E. coli* cell wall composed of an outer membrane containing lipopolysaccharides, a periplasmic space with a peptidoglycan layer which may prevent penetration of the highly non-polar lauryl gallate. Alkyl (C-4 and C-5) gallates at concentrations lower than their MIC effectively increased the sensitivity of oxacillin against MRSA [38], with the amphiphilic properties of alkyl gallates enabling them to interact with the *S. aureus* cell wall. The galloyl moiety plays a key role in β-lactam activity intensification. The structure of the alkyl gallates enables them to interact with many targets in the bacterial cell wall, which disrupts the structure of the cell wall and renders the process of cell wall synthesis inefficient. Of note is that octyl gallate, with a C-8 alkyl chain length, significantly increased the antimicrobial activity of bacitracin against multiple drug resistant *S. aureus* [38].

The gram-positive and sphere–shaped *S. aureus* bacteria were selected for the study on the morphologic effect of gallic acid and its analogues. It is the most dangerous of all the staphylococcal bacteria which can cause skin, bloodstream, lung, and medical implant infections, endocarditis, and osteomyelitis [39]. Gallic acid and ethyl gallate are both shown here to apparently affect the permeability of the *S. aureus* cell wall, with extrusion of cell contents leading to an apparent collapse in cell shape. The effect of gallic acid appears more prominent than that of ethyl gallate within the first hour of incubation, consistent with previous finding [40]. Binding to lipids by the alkyl gallates generally increases with increasing alkyl chain length. Self-association and membrane binding of the alkyl gallates might therefore be primary factors associated with their antibacterial activities [41]. Alkyl gallates also disrupt the cell division protein FtsZ-ring in *B. subtilis* cells, resulting in cell elongation. In vitro, alkyl gallates cause FtsZ protein to cluster and lose its capacity to polymerize through their very tight binding to FtsZ. They also target *B. subtilis* membrane integrity [42]. Our results share some agreements with these findings. These novel alkyl (C-4 and C-5) gallates can effectively disrupt the cell wall integrity of *S. aureus*, which may offer a potentially exploitable synergistic activity with some antibiotics to target resistant bacterial cells with impermeable cell wall or efflux pump proteins. Plant-derived polyphenols have been shown to inhibit bacterial growth [43] and formation of biofilms, an important factor causing antibacterial resistance [4]. Therefore, the presence of various polyphenols in the most active fraction should also contribute to its overall antibacterial activity.

## Conclusion

In summary, bioassay-guided fractionation of *C. gabunensis* bark extract yielded an active antibacterial fraction. Using GC-MS, LC-MS and NMR techniques, gallic acid (**1**) and ethyl gallate (**6**) and other putative polyphenols were identified and likely contributed to the antibacterial activity of *C. gabunensis* extract. Synthesis of four alkyl gallates and an alkenyl gallate was carried out. Preliminary SAR of gallate derivatives indicates that increasing the length of the alkyl chain of alkyl gallate generally increases their antibacterial potency. The results of this study support the ethnobotanical use of *C. gabunensis* and suggest that ethyl gallate and other polyphenols be potential antibacterial agents.

## Additional file

Additional file 1: **Figure S1.** scheme summarizing the bioassay-guided isolation procedure for *C. gabunensis* bark. **Figure S2.** agar plate showing the effect of CGE against *Staphylococcus epidermidis*. **Table S1.** antibacterial activity of *Cylicodiscus gabunensis* extracts and fractions. **Table S2.** antibacterial activity of ampicillin as positive control using disk diffusion assay. **Table S3.** MIC (μg/ml) of sub-fractions CGEEA-F5-(1–12). **Figure S4.** GC-MS chromatogram of *Cylicodiscus gabunensis* CGEEA-F5. **Figure S5.** GC-MS chromatogram of CGEEA-F5–8. **Figure S6.** EI-mass spectra of compounds detected in CGEEA-F5–8. **Table S4.** compounds (after TMS derivation) detected from CGEEA-F5 by GC-MS. **Figure S7.** analytical HPLC of CGEEA-F5–8. **Figure S8.** A: LC-MS chromatogram of CGEEA-F5–8. B: positive ESI-MS of ethyl gallate. **Figure S9.** ^1^H NMR (300 MHz, CD_3_OD) of CGEEA-F5–8, and 0.9. (DOCX 2095 kb)

## Data Availability

All data generated or analysed during this study are included in this published article and its supplementary information files.
