# Supplementary material for: Discovery, synthesis and antibacterial evaluation of phenolic compounds from Cylicodiscus gabunensis
Source: BMC Complement Altern Med. 2019 Jul 24;19:183. doi: 10.1186/s12906-019-2589-2 (PMC6651987; doi:10.1186/s12906-019-2589-2)
Supplement: Supplementary file 1 — Figure S1. scheme summarizing the bioassay-guided isolation procedure for C. gabunensis bark. Figure S2. agar plate showing the effect of CGE against Staphylococcus epidermidis. Table S1. antibacterial activity of Cylicodiscus gabunensis extracts and fractions. Table S2. antibacterial activity of ampicillin as positive control using disk diffusion assay. Table S3. MIC (μg/ml) of sub-fractions CGEEA-F5-(1–12). Figure S4. GC-MS chromatogram of Cylicodiscus gabunensis CGEEA-F5. Figure S5. GC-MS chromatogram of CGEEA-F5–8. Figure S6. EI-mass spectra of compounds detected in CGEEA-F5–8. Table S4. compounds (after TMS derivation) detected from CGEEA-F5 by GC-MS. Figure S7. analytical HPLC of CGEEA-F5–8. Figure S8. A: LC-MS chromatogram of CGEEA-F5–8. B: positive ESI-MS of ethyl gallate. Figure S9. 1H NMR (300 MHz, CD3OD) of CGEEA-F5–8, and 0.9. (DOCX 2095 kb) [file 12906_2019_2589_MOESM1_ESM.docx]

**Supplementary Materials**

**Discovery, synthesis and antibacterial evaluation of phenolic compounds from Cylicodiscus gabunensis Harms**

Omar Aldulaimi^1, 2^, Falko Drijfhout,^3^ Fidelia I. Uche^1^, Paul Horrocks^1^, Wen-Wu Li^1*^

^1^School of Pharmacy and Bioengineering, Keele University, United Kingdom

^2^College of Pharmacy, Al-Mustansiriyah University, Iraq

^3^Chemical Sciences Research Centre, Keele University, United Kingdom

**Corresponding author:**

Dr. Wen-Wu Li

E-mail: w.li@keele.ac.uk

Phone: +44 (0)1782 674382

Fax: +44 (0)1782 747319

**Table S1:** Antibacterial activity of *Cylicodiscus gabunensis* extracts and fractions (n=3). NC: not counted. Neg: negative results (the extract did not kill the bacteria). NA: not assayed.

| Bacteria strain (gram stain) | Extract | Mean diameter of IZ (mm) ± SD (n =3) | | | | | | |
| --- | --- | --- | --- | --- | --- | --- | --- | --- |
|  |  | 2 mg/disk | 1 mg/disk | 0.5 mg/disk | 0.25 mg/disk | 0.5 mg/disk | | |
|  |  |  |  |  |  | Polar extract fractions | | |
|  |  |  |  |  |  | CGEEA | CGEBU | CGEAQ |
| *Staphylococcus aureus* (+) | CGH | 7.5 ± 0.5 | 7.0 ± 0.4 | NC | NC | 14.0±0.4 | 11.5±1.5 | 7.5±0.4 |
|  | CGE | 16.5 ± 0.5 | 12.0 ± 1.0 | 10.5±0.5 | 7.5±0.6 |  |  |  |
| *Staphylococcus epidermidis* (+) | CGH | Neg. | Neg. | Neg. | Neg. | 15.0±1.0 | 10.5±0.6 | 11.5±0.6 |
|  | CGE | 19.5±1.0 | 14.5±0.5 | 13.5±0.5 | 10.0±1.0 |  |  |  |
| *Bacillus cereus* (+) | CGH | Neg. | Neg. | Neg. | Neg. | 11.0±0.4 | 8.0±0.5 | 8.5±0.5 |
|  | CGE | 12.5±0.6 | 11.0±1.0 | 10.0±0.5 | 8.0±1.0 |  |  |  |
| *Bacillus subtilis* (+) | CGH | Neg. | Neg. | Neg. | Neg. | NA | NA | NA |
|  | CGE | 10.6±0.5 | 8.5±0.5 | 7.0±0.5 | Neg. |  |  |  |
| *Streptococcus faecalis* (+) | CGH | Neg. | Neg. | Neg. | Neg. | NA | NA | NA |
|  | CGE | 10.5±0.5 | 8.0±0.5 | 7.0±0.5 | Neg. |  |  |  |
| *Escherichia coli* (-) | CGH | Neg. | Neg. | Neg. | Neg. | NA | NA | NA |
|  | CGE | Neg. | Neg. | Neg. | Neg. |  |  |  |
| *Enterobacter cloacae* (-) | CGH | Neg. | Neg. | Neg. | Neg. | NA | NA | NA |
|  | CGE | Neg. | Neg. | Neg. | Neg. |  |  |  |
| *Pseudomonas aeruginosa* (-) | CGH | Neg. | Neg. | Neg. | Neg. | NA | NA | NA |
|  | CGE | Neg. | Neg. | Neg. | Neg. |  |  |  |
| *Alcaligenes faecalis* (-) | CGH | 9.5 ± 0.5 | 7.5 ± 0.5 | Neg. | Neg. | 16.0±1.0 | 13.0±0.5 | 11.5±1.0 |
|  | CGE | 17.0 ± 2.0 | 15.6 ± 1.4 | 15.0 ± 1.0 | 11.0 ±2.0 |  |  |  |

**Table ‎S2:** Antibacterial activity of ampicillin as positive control using disk diffusion assay. Neg: negative results (the antibiotic did not kill the bacteria). NA: not assayed.

| Bacteria strains  (gram stain) | Mean diameter of IZ (mm) ± SD (n =3) | |
| --- | --- | --- |
|  | Ampicillin  10 µg/disk | Ampicillin  2 µg/disk |
| *Staphylococcus aureus* (+) | NA | 32±3.0 |
| *Staphylococcus epidermidis* (+) | NA | 36±2.5 |
| *Bacillus cereus* (+) | Neg. | NA |
| *Bacillus subtilis* (+) | NA | 28±1.0 |
| *Streptococcus faecalis* (+) | NA | 24±2.0 |
| *Escherichia coli* (-) | 22±2.0 | 16±1.0 |
| *Enterobacter cloacae* (-) | 24±1.0 | 17±1.5 |
| *Pseudomonas aeruginosa* (-) | NA | NA |
| *Alcaligenes faecalis* (-) | 10±1.0 | NA |

Table S3: MIC (µg/ml) of sub-fractions CGEEA-F5-(1-12).

| Fraction | *Bacterial isolate* | | | |
| --- | --- | --- | --- | --- |
|  | *S. aureus* | *S. epidermidis* | *E. coli* | *A. faecalis* |
| CGEEA-F5-1 | ˃512 | ˃ 512 | ˃512 | ˃512 |
| CGEEA-F5-2 | NA | ˃512 | NA | NA |
| CGEEA-F5-3 | NA | ˃512 | NA | NA |
| CGEEA-F5-4 | NA | ˃512 | NA | NA |
| CGEEA-F5-5 | NA | ˃512 | NA | NA |
| CGEEA-F5-6 | NA | ˃512 | NA | NA |
| CGEEA-F5-7 | 256 | 256 | ˃512 | 512 |
| CGEEA-F5-8 | 128 | 64 | 512 | 128 |
| CGEEA-F5-9 | 256 | 128 | ˃512 | 512 |
| CGEEA-F5-10 | ˃512 | 512 | 512 | ˃512 |
| CGEEA-F5-11 | ˃512 | ˃512 | 512 | ˃512 |
| CGEEA-F5-12 | ˃512 | ˃512 | ˃512 | ˃512 |

NA, not assayed

| Peak no. | RT  (min) | Compound directly detected by GC-MS | The parent compounds | percentages ±SD (n=3) | RI |
| --- | --- | --- | --- | --- | --- |
| 1 | 10.57 | 1,3-bis(1,1-dimethylethyl)- Benzene | 1,3-bis(1,1-dimethylethyl)- Benzene | 8.96 ±0.45 | 1262 |
| 2 | 13.24 | Benzene, 1-(trimethylsilyloxy)-2-(trimethylsilyloxymethyl)- | 2-(Hydroxymethyl)phenol or salicylic alcohol | 9.60 ±0.34 | 1442 |
| 3 | 14.45 | 4-Ethoxy-benzoic acid ethyl ester | 4-Ethoxy-benzoic acid ethyl ester | 6.99 ±1.01 | 1533 |
| 4 | 15.72 | Benzoic acid, 4-[(trimethylsilyl)oxy]-, trimethylsilyl ester | 4-hydroxybenzoic acid | 3.97 ±0.01 | 1634 |
| 5 | 18.03 | Benzoic acid, 3,4-bis[(trimethylsilyl)oxy]-, trimethylsilyl ester | 3,4-dihydroxybenzoic acid or (protocatechuic acid) | 10.45 ±1.20 | 1836 |
| 6 | 18.69 | Trimethylsilyl 3,5-dimethoxy-4-(trimethylsilyloxy)benzoate | 3,5-dimethoxy-4-hydroxy-benzoic acid or syringic acid | 5.73 ±0.70 | 1892 |
| 7 | 19.37 | 3,4,5-Trihydroxybenzoic acid ethyl ester, tris(O-trimethylsilyl)- | 3,4,5-trihydroxybenzoic acid ethyl ester or ethyl gallate | 8.97±0.80 | 1960 |
| 8 | 19.55 | Benzoic acid, 3,4,5-tris(trimethylsiloxy)-, trimethylsilyl ester | 3,4,5-trihydroxy benzoic acid or = gallic acid | 22.30 ±3.56 | 1973 |
| 9 | 20.27 | Hexadecanoic acid, trimethylsilyl ester | Hexadecanoic acid | 7.56 ±0.35 | - |
| 10 | 22.16 | Octadecanoic acid, trimethylsilyl ester | Octadecanoic acid | 9.98 ±1.23 | - |

**Table S4:** Compounds (after TMS derivation) detected from CGEEA-F5 by GC-MS. RT = Retention time, RI= Retention index.


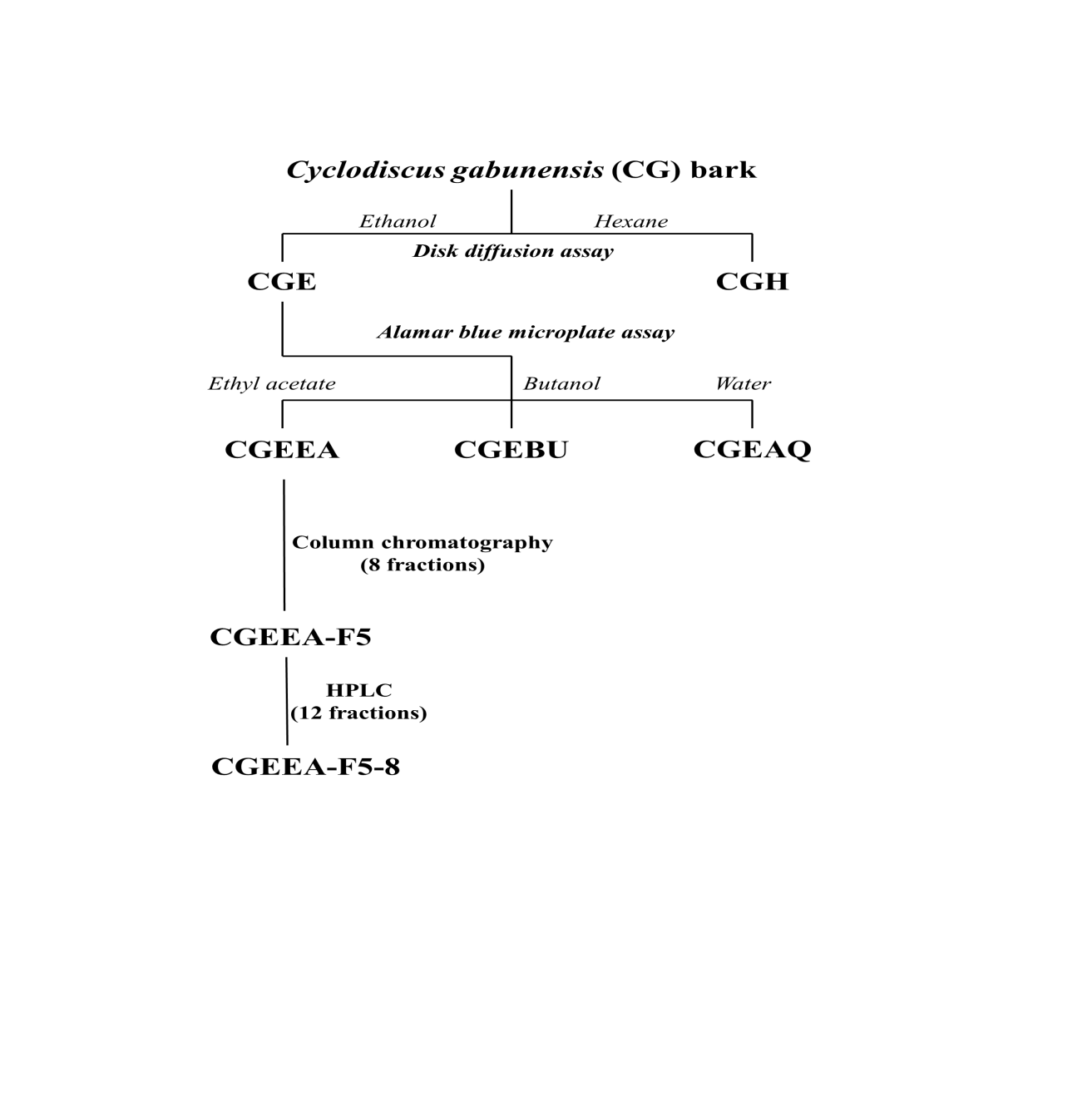


Figure S1: Scheme summarizing the bioassay-guided isolation procedure for *C. gabunensis* bark.


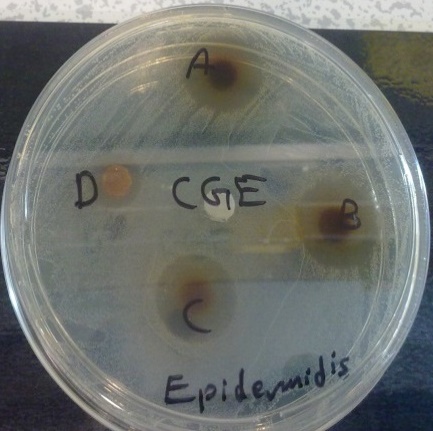


**IZ**

Figure S2: Agar plate shows the effect of CGE against *Staphylococcus epidermidis* concentration of CGE (A) 2mg/disk, (B) 1mg/disk, (C) 0.5 mg/disk and (D) 0.25 mg/disk. The clear inhibition zone (IZ) for 0.5mg/disk of CGE is indicated by the arrow.


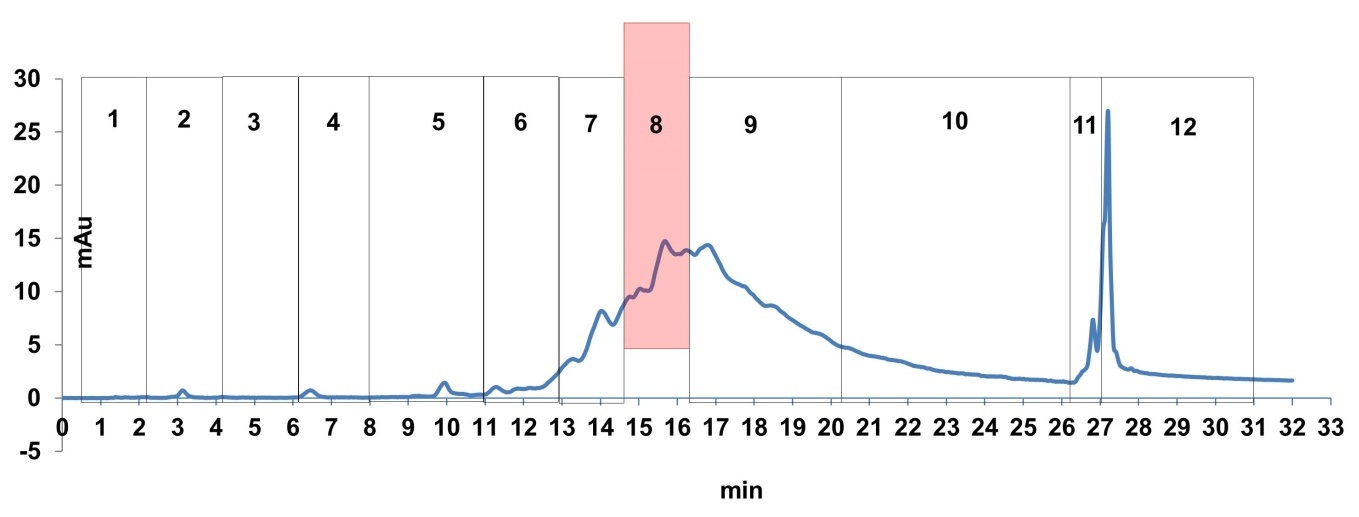


**Figure S‎3:** Preparative HPLC of CGEEA-F5. Sub-fractions were collected according to defined time intervals.


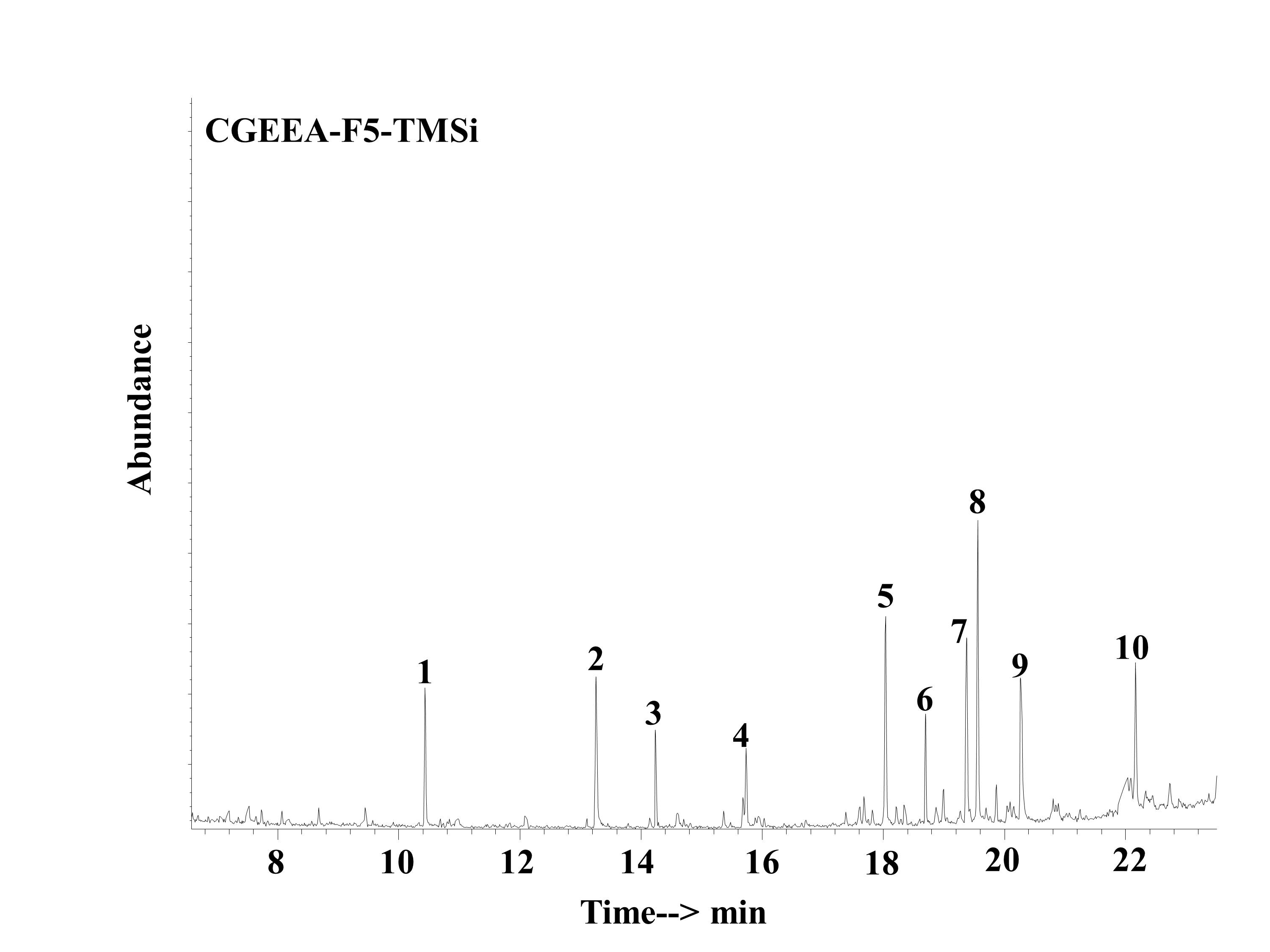


**Figure S4:** GC-MS chromatogram of *Cylicodiscus gabunensis* CGEEA-F5.

The analytes were derived to TMSi derivatives, and analysed in triplicate, n=3. Identification of compounds 1-10 is shown in Table S4.


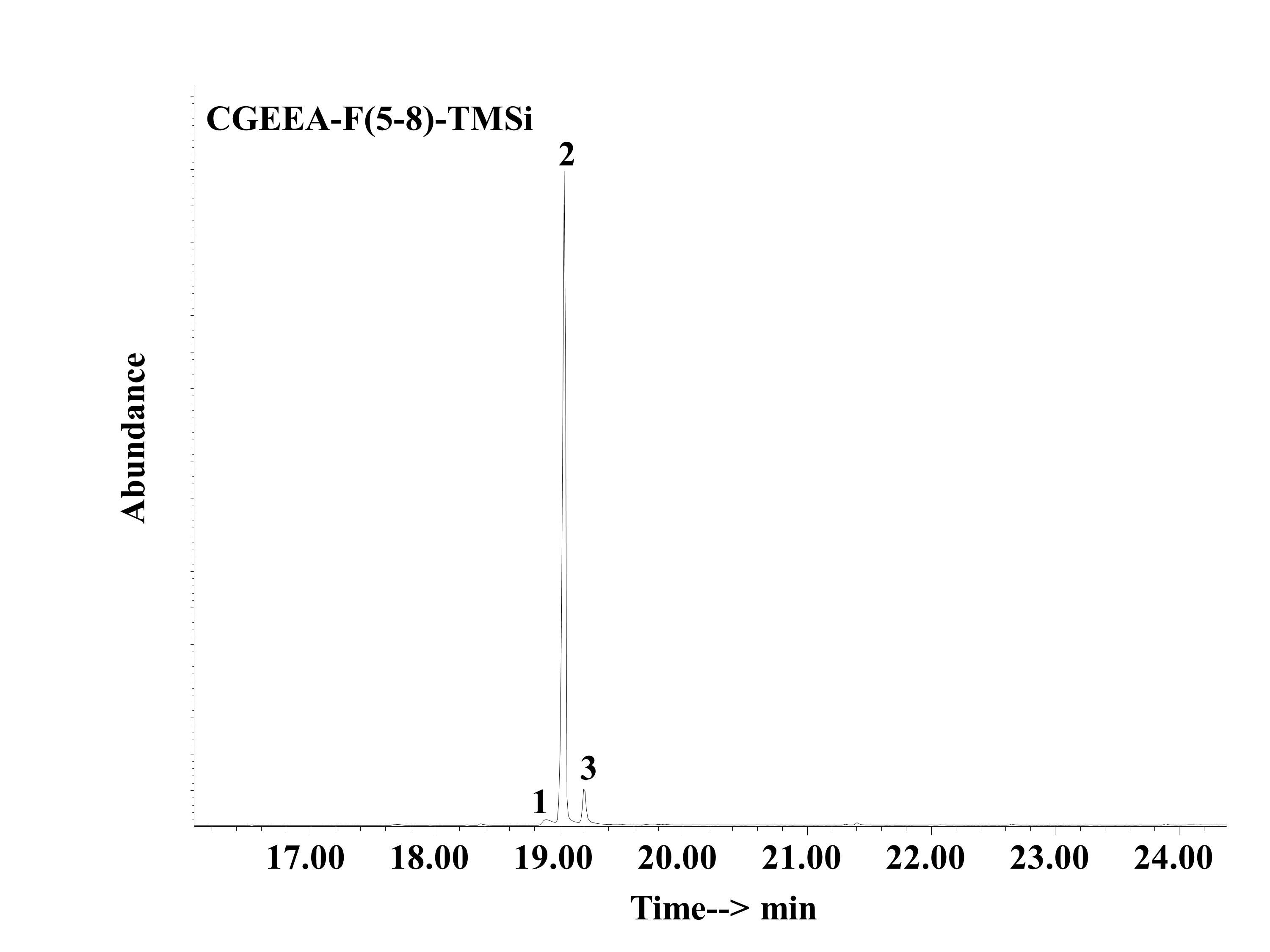


**Figure S5:** GC-MS chromatogram of CGEEA-F5-8. Peak 1: syringic acid-TMSi; peak 2: ethyl gallate-TMSi; peak 3: gallic acid-TMSi. The fraction was analysed in triplicate, n=3.


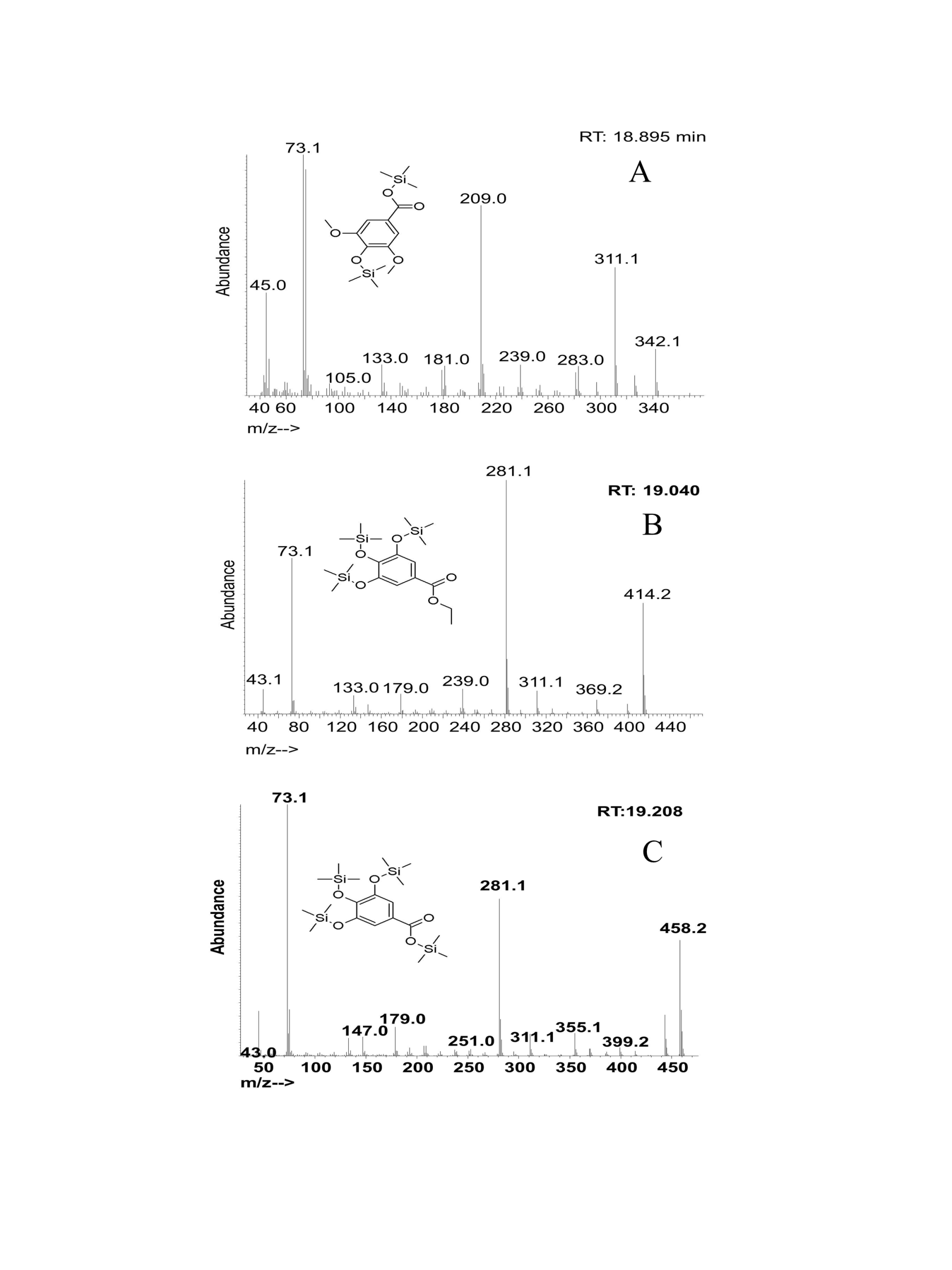


**Figure ‎S6:** EI-Mass spectra of compounds detected in CGEEA-F5-8. A: syringic acid-TMSi; B: ethyl gallate-TMSi; C: gallic acid-TMSi.


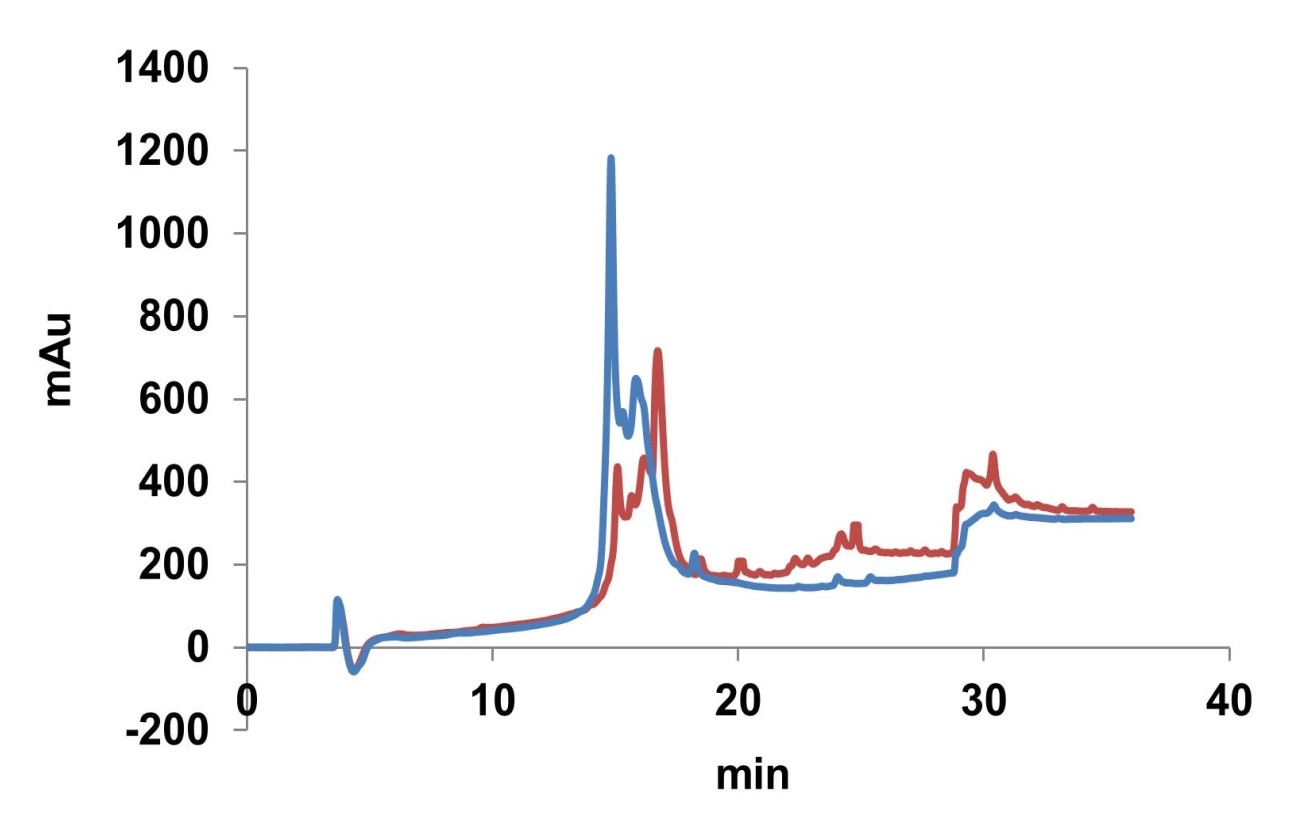


Figure S7: Analytical HPLC of CGEEA-F5-8. The black arrow refers to the spiked ethyl gallate.


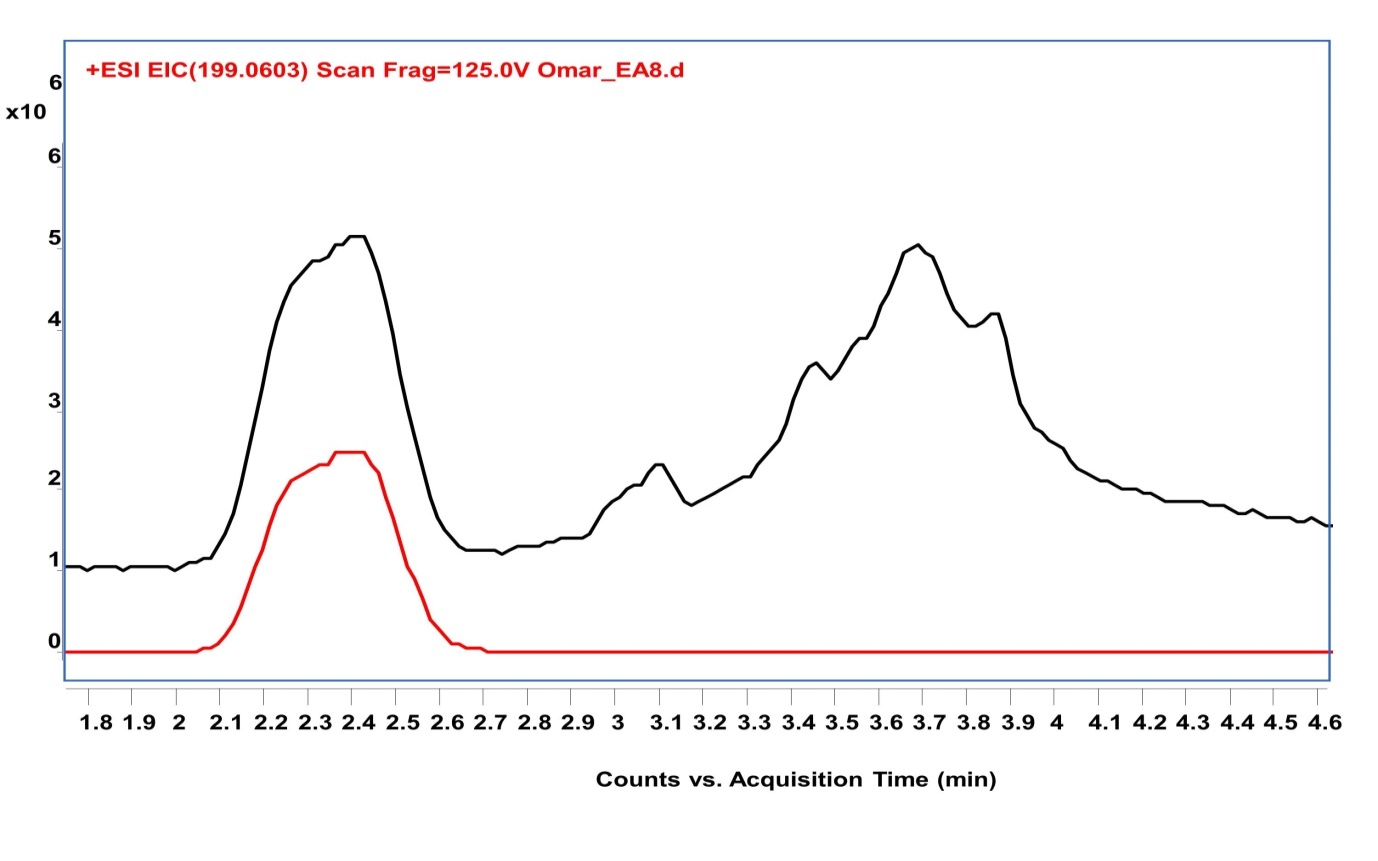

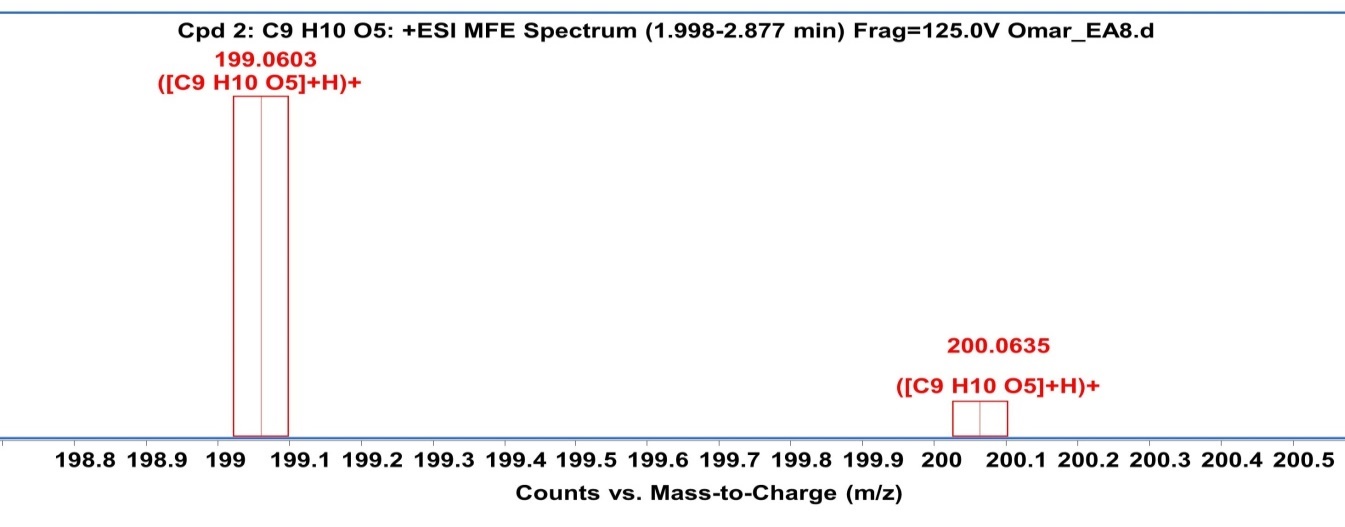


**A**

**B**

Figure S8: A: LC-MS chromatogram of CGEEA-F5-8. B: positive ESI-MS of ethyl gallate.


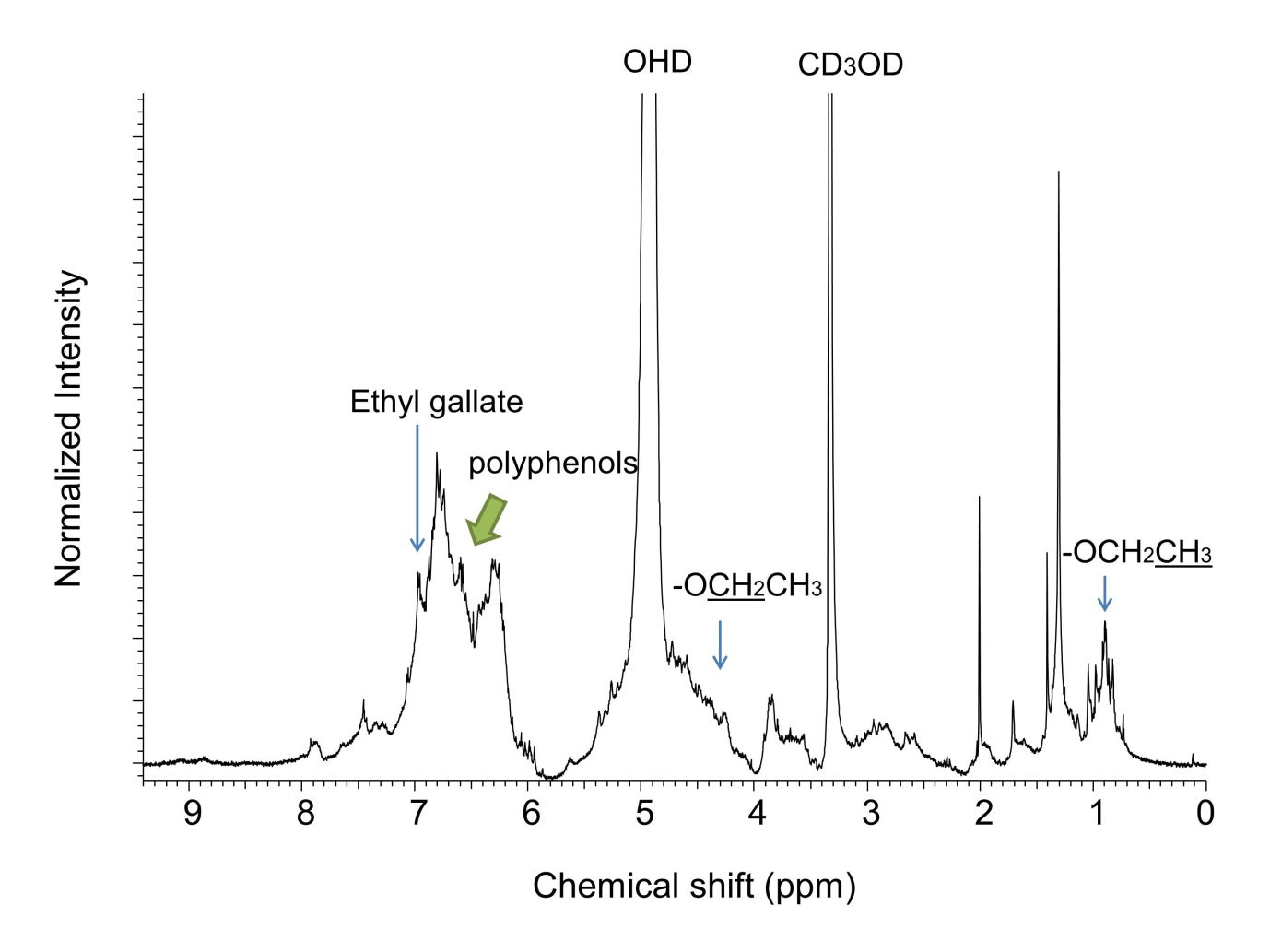


Figure S9: ^1^H NMR (300 MHz, CD_3_OD) of CGEEA-F5-8. The aromatic protons and ethyl group of ethyl gallate are indicated around 7.0, 4.2/0.9 ppm, respectively. The complex signals between 6-8 ppm and 3-5 ppm indicate the presence of a complex mixture of polyphenols (Table S5).
